# Posterior fixation can further improve the segmental alignment of lumbar degenerative spondylolisthesis with oblique lumbar interbody fusion

**DOI:** 10.1186/s12891-021-04086-y

**Published:** 2021-02-23

**Authors:** Jingye Wu, Tenghui Ge, Ning Zhang, Jianing Li, Wei Tian, Yuqing Sun

**Affiliations:** Department of Spine Surgery, Beijing Jishuitan Hospital, No. 31, Xinjiekou East Street, Xicheng District, Beijing, 100035 People’s Republic of China

**Keywords:** Spondylolisthesis, Interbody fusion, OLIF, Sagittal alignment, Slip reduction, Segmental lordosis

## Abstract

**Background:**

For patients with degenerative spondylolisthesis, whether additional posterior fixation can further improve segmental alignment is unknown, compared with stand-alone cage insertion in oblique lumbar interbody fusion (OLIF) procedure. The aim of this study was to compare changes of the radiographical segmental alignment following stand-alone cage insertion and additional posterior fixation in the same procedure setting of OLIF for patients with degenerative spondylolisthesis.

**Methods:**

A retrospective observational study. Selected consecutive patients with degenerative spondylolisthesis underwent OLIF procedure from July 2017 to August 2019. Five radiographic parameters of disc height (DH), DH-Anterior, DH-Posterior, slip ratio and segmental lordosis (SL) were measured on preoperative CT scans and intraoperative fluoroscopic images. Comparisons of those radiographic parameters prior to cage insertion, following cage insertion and following posterior fixation were performed.

**Results:**

A total of thirty-three patients including six males and twenty-seven females, with an average age of 66.9 ± 8.7 years, were reviewed. Totally thirty-six slipped levels were assessed with thirty levels at L4/5, four at L3/4 and two at L2/3. Intraoperatively, with only anterior cage support, DH was increased from 8.2 ± 1.6 mm to 11.8 ± 1.7 mm (*p* < 0.001), DH-Anterior was increased from 9.6 ± 2.3 mm to 13.4 ± 2.1 mm (*p* < 0.001), DH-Posterior was increased from 6.1 ± 1.9 mm to 9.1 ± 2.1 mm (*p* < 0.001), the slip ratio was reduced from 11.1 ± 4.6% to 8.3 ± 4.4% (*p* = 0.020) with the slip reduction ratio 25.6 ± 32.3%, and SL was slightly changed from 8.7 ± 3.7° to 8.3 ± 3.0°(*p* = 1.000). Following posterior fixation, the DH was unchanged (from 11.8 ± 1.7 mm to 11.8 ± 2.3 mm, *p* = 1.000), DH-Anterior and DH-Posterior were slightly changed from 13.4 ± 2.1 mm and 9.1 ± 2.1 mm to 13.7 ± 2.3 mm and 8.4 ± 1.8 mm respectively (*P* = 0.861, *P* = 0.254), the slip ratio was reduced from 8.3 ± 4.4% to 2.1 ± 3.6% (*p* < 0.001) with the slip reduction ratio 57.9 ± 43.9%, and the SL was increased from 8.3 ± 3.0° to 10.7 ± 3.6° (*p* = 0.008).

**Conclusions:**

Compared with stand-alone cage insertion, additional posterior fixation provides better segmental alignment improvement in terms of slip reduction and segmental lordosis in OLIF procedures in the treatment of lumbar degenerative spondylolisthesis.

## Background

Lumbar degenerative spondylolisthesis is the anterior slip of one vertebral body on another in the presence of an intact neural arch [1] with underlying pathologies of segmental instability, anterior slip and spinal stenosis [2, 3]. Neural decompression and segmental stabilization are aims of surgical treatment, thus traditional surgical techniques involve direct neural decompression and instrumented fusion. As a recently developed minimally invasive technique for degenerative lumbar disease [4, 5], oblique lumbar interbody fusion (OLIF) can provide both direct and indirect neural decompression and segmental fusion, and gains the popularity in surgical treatment of degenerative spondylolisthesis.

Inserting a large cage into the intervertebral space, OLIF procedure can enlarge the canal and achieve indirect neural decompression by decreasing disc bulge and folds of ligamentum flavum [6]. Two techniques of OLIF procedure for degenerative spondylolisthesis were described in the literature, cage insertion with or without posterior fixation (stand-alone technique). Shortening surgical time without the cost of posterior fixation, stand-alone technique for selected patients is favored by some surgeons [7, 8]. However, the cage subsidence, migration and relatively high reoperation rate were reported [9, 10]. The additional posterior fixation can provide more stability during the course of bony fusion without those complications of stand-alone technique [9].

Another advantage of posterior fixation lies on segmental alignment improvement. For mild to moderate slip degree of spondylolisthesis, inserting cage alone can partially reduce the slip due to tightening the surrounding ligamentous structures, thus improve the segmental alignment which may be further improved by additional posterior fixation with reduction maneuver. To the authors’ knowledge, whether additional posterior fixation can further improve segmental alignment than did the stand-alone technique is unknown in the literature. The aim of this study is to assess the changes of segmental alignment following stand-alone cage insertion and additional posterior fixation in the same procedure setting for degenerative spondylolisthesis.

## Material and methods

### Patients population

Consecutive patients who had degenerative spondylolisthesis from L1 to L4 and underwent OLIF procedure with posterior pedicle screw fixation from July 2017 to August 2019 in the authors’ hospital were retrospectively reviewed. Surgical indications for those patients were symptomatic radiculopathy or neurological claudication that were unresponsive to at least 3 months conservative treatments. Persistent mechanical low back pain and signs indicating segmental instability together with hypermobility suggested by flexion-extension lateral radiographs were confirmed before surgeries. Patients with isthmic spondylolisthesis, high grade spondylolisthesis (greater than Meyerding Grade 2 spondylolisthesis), degenerative scoliosis (Cobb angle > 30°) and L5 degenerative spondylolisthesis were excluded.

Selected patients underwent OLIF procedure with percutaneous pedicle screw fixation for indirect decompression. Indications for indirect decompression include: intermittent neurological symptoms can be resolved by lying down and rest; <Grade 4 facet joint osteoarthritis [11]; no bony lateral recess stenosis; no non-contained disc.

A total of 33 patients including 6 males and 27 females, with an average age of 66.9 ± 8.7 years, were selected in this study. Twenty-one patients underwent one-level fusion, 9 two-level fusion and 3 multi-level (≥three levels) fusion. Totally 36 slipped levels were assessed in this study. Preoperative slip ratio was 11.1 ± 4.6% (Range: 3.5 to 23.0%) on average. Eleven patients received indirect decompression and percutaneous pedicle screw fixation. The detailed characteristics of patients were shown in Table 1. All these selected patients have mid-term follow-up (postoperative 1 year) with intact pain and disability scores. Twenty-nine patients had postoperative plain and flexion-extension views of lumbar spine at postoperative 1 year. The study was approved by the ethical committee of the authors’ hospital.

**Table 1:** Patient characteristics

No. of patients	33
Age (years)	66.9 ± 8.7
Female: Male	27:6
Fusion levels	
One-level fusion	20
Two-level fusion	10
Multi-level fusion(≥3 levels fusion)	3
Slipped level	
L2/3	2
L3/4	4
L4/5	30
Preoperative slip ratio (%)	11.1 ± 4.6
Average surgical time (minutes)	199 ± 49
Estimated blood loss (ml)	209 ± 99
Indirect decompression with percutaneous pedicle screw fixation	11 (12 levels)

### Radiographic parameter measures

**Fig. 1:**
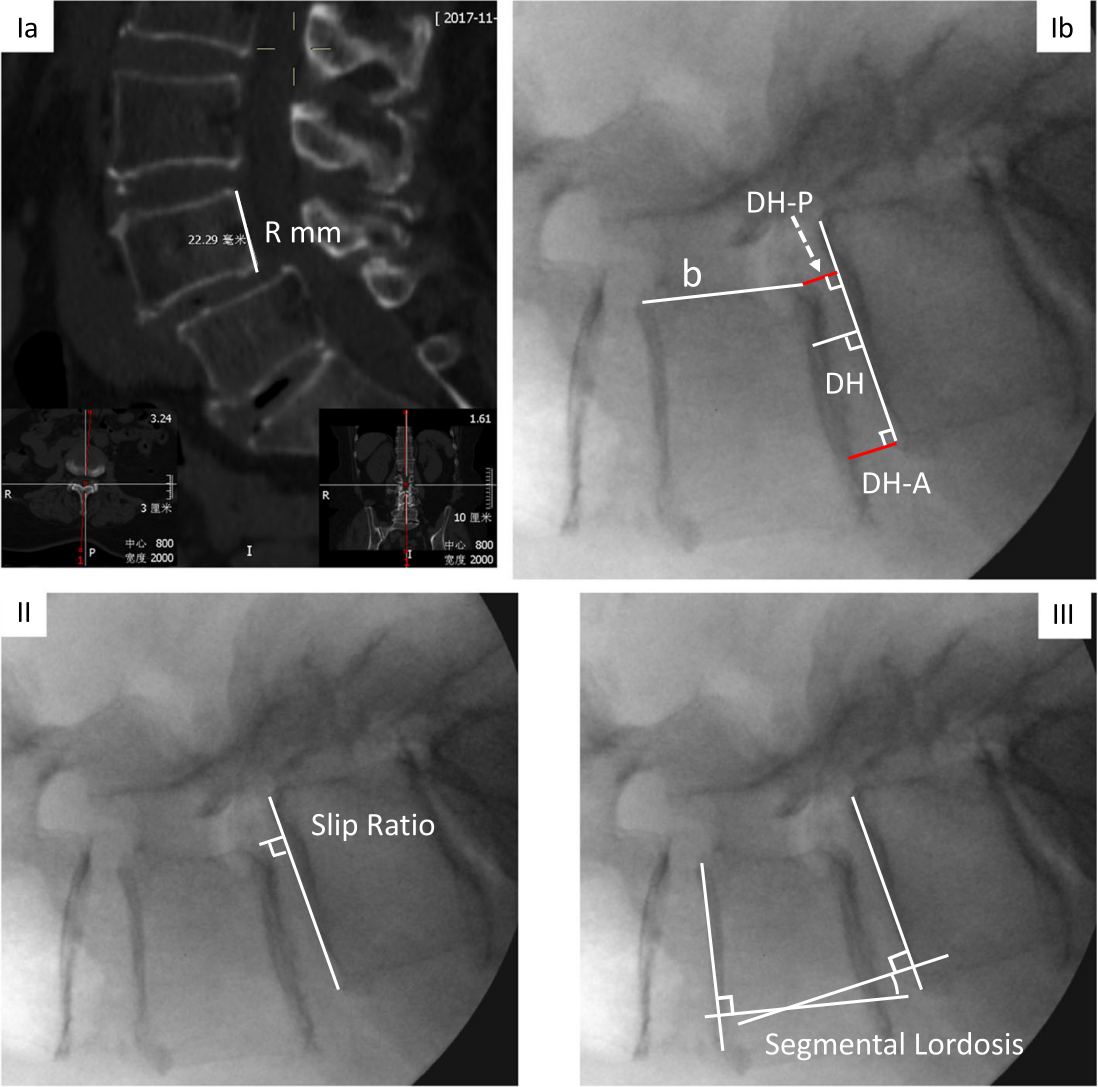
The methods of measurement for Disc Height (DH), slip ratio and Segmental Lordosis (SL). **Ia.** The length of posterior wall of L4 vertebral body were chosen as a reference, R mm. **Ib.** Draw a perpendicular line from the midpoint of superior endplate of L5. The distance between midpoint of L5 superior endplate and intersection point of this perpendicular line and L4 inferior endplate was measured as a mm. Measure the length of posterior wall of L4 vertebral body as b mm. DH was R × a/b mm. DH-A and DH-P were anterior and posterior distances of the overlapped disc space. **II.** draw a perpendicular line from the posteroinferior point of L4 vertebral body to the L5 superior endplate and measure the distance of intersection point and posterosuperior point of L5 vertebral body as c mm. The slip ratio defined as the ratio of c to the length of L5 superior endplate. **III**. angulation between the lines of L4 superior endplate and L5 superior endplate

Five radiographic parameters including disc height (DH), DH-Anterior, DH-Posterior, slip ratio and segmental lordosis (SL) were used to assess the segmental alignment of index level which were measured on the lateral views of fluoroscopic images that were obtained prior to cage insertion, following cage insertion and following posterior fixation. Taking L4/5 degenerative spondylolisthesis for example, the definition and measurement methods for radiographic parameters of interest are delineated as follows and illustrated on Fig. 1.

- Disc height (DH): Due to the inconsistency of fluoroscopic magnification, disc height cannot be measured directly on fluoroscopic images. The length of posterior wall of L4 vertebral body on the midsagittal cut of preoperative CT scans were chosen as a reference distance, R mm. A perpendicular line was drawn from the midpoint of superior endplate of L5 on the lateral fluoroscopic view. The distance between midpoint of L5 superior endplate and intersection point of this perpendicular line and L4 inferior endplate was measured as a mm. The length of posterior wall of L4 vertebral body was measured and recorded as b mm on the lateral fluoroscopic view. DH was defined as R × a/b mm. DH-Anterior was defined as the vertical distance from posteroinferior corner of L4 vertebral body to superior endplate of L5. DH-Posterior was defined as the vertical distance from anterosuperior of L5 to the inferior endplate of L4.
- Slip ratio: A perpendicular line was drawn from the posteroinferior point of L4 vertebral body to the L5 superior endplate and the distance of from the intersection point to posterosuperior point of L5 vertebral body was measured and recorded as c mm on the lateral fluoroscopic view. The slip ratio was defined as the ratio of c to the length of L5 superior endplate.
- Slip reduction ratio: defined as the change of slip ratio following cage insertion or posterior fixation divided by slip ratio prior to cage insertion.
- Segmental lordosis (SL): defined as angulation between the parallel lines of L4 superior endplate and L5 superior endplate on the lateral fluoroscopic view.

Preoperative CT scans and intraoperative fluoroscopic images were transferred into Carestream PACS (Version 11.0) and OsiriX Lite (Version 10.0.5) respectively. To ensure reliability of measurement, two observers received training of measurement on software workstation and the inter-observer reliability were assessed by interclass correlation coefficient (ICC). The ICC value were greater than 0.75 which indicated good reliability of measurement, and the average value of two observations was calculated for statistical analysis.

### OLIF procedure

After general anesthesia, the patient was placed in right decubitus position. The center of intervertebral disc of the index level was identified under fluoroscopy and marked on skin. A skin incision was made 4–10 cm anterior to the center of index intervertebral disc. The musculature of abdominal wall was bluntly divided along the muscle fibers until the retroperitoneal space was reached. The psoas major and abdominal aorta were palpated and bluntly dissected through the interval between them by the surgeon’s fingers. The tip of guiding wire was placed at the intervertebral space and confirmed under fluoroscopy. Afterwards, a serial of dilators was placed and the final pathway was established. The disc was incised and removed, followed by endplate preparations, implant trialing and grafting. Appropriate size of cage with 6 degrees of lordosis (Clydesdale Spinal System, Medtronic) was chosen and inserted into proper position which was confirmed under fluoroscopy.

Percutaneous pedicle screw fixation was performed in selected patients who met the criteria for indirect decompression, which was guided by computed navigation system or robotic system through stab incisions. Percutaneous reduction was performed following the manuals of the provider (Viper MIS Spine System, DePuy Spine). A pistol-grip reducer was used to apply internal reduction for the cranial screw after the caudal screws were tightened. If direct decompression was planned, posterior midline dissection and exposure was performed, followed by pedicle screw fixation and partial laminectomy. Reduction maneuvers by screw-rod construct were attempted for all cases. Following rod bending and tightening the caudal screw heads, the cranial screw heads were gradually tightened during reduction maneuver. No compressive force across pedicle screw heads to increase segmental lordosis was applied for all patients.

### Statistical analysis

Statistical analysis was performed using SPSS Version 23.0 (IBM Corp, Chicago, Illinois). Categorical data were presented as numbers and/or ratio, while numerical data as mean and standard deviation. Statistical significance level was defined as *P* < 0.05 on the basis of two-sided hypothesis test.

One-way ANOVA was used to compare the radiographic parameters prior to cage insertion, following cage insertion and following reduction maneuver. Multiple comparisons were performed for radiographic parameters prior to cage insertion and following cage insertion, as well as following cage insertion and following posterior fixation if one-way ANOVA result was statistically significant. Sub-group analysis was performed to compare the effect of open and percutaneous techniques of pedicle screw fixation on radiographic parameters by using ANOVA with repeated measures and Student *t* test. Student paired *t*-test was used to compare the preoperative and postoperative pain and disability scores to assess the clinical improvement.

## Results

Intraoperatively, with only anterior cage support, DH was increased from 8.2 ± 1.6 mm to 11.8 ± 1.7 mm (*p* < 0.001), DH-Anterior was increased from 9.6 ± 2.3 mm to 13.4 ± 2.1 mm (*p* < 0.001), DH-Posterior was increased from 6.1 ± 1.9 mm to 9.1 ± 2.1 mm (*p* < 0.001), the slip ratio was reduced from 11.1 ± 4.6% to 8.3 ± 4.4% (*p* = 0.020) with the slip reduction ratio 25.6 ± 32.3%, and SL was unchanged (from 8.7 ± 3.7° to 8.3 ± 3.0°, *p* = 1.000).

**Fig. 2:**
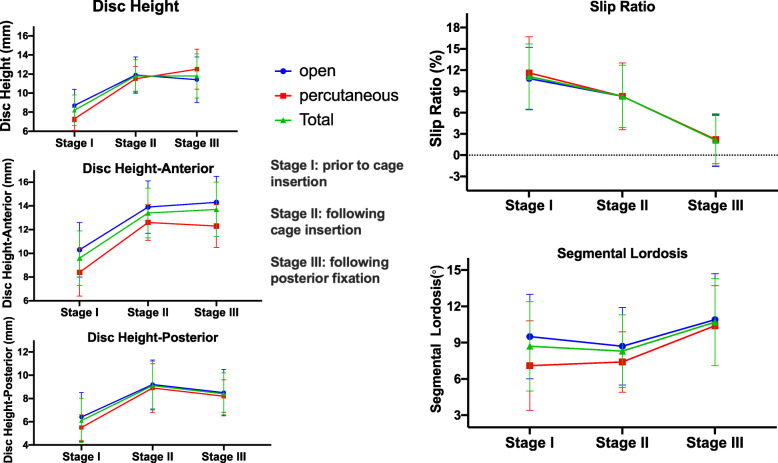
Changes of radiographic parameter (DH, DH-Anterior, DH-Posterior, SL and slip ratio) at different stages of OLIF procedures. The values were expressed as means and standard deviations

Following posterior fixation, the DH was unchanged (from 11.8 ± 1.7 mm to 11.8 ± 2.3 mm, *p* = 1.000), DH-Anterior and DH-Posterior were slightly changed from 13.4 ± 2.1 mm and 9.1 ± 2.1 mm to 13.7 ± 2.3 mm and 8.4 ± 1.8 mm respectively (*P* = 0.861, *P* = 0.254), the slip ratio was reduced from 8.3 ± 4.4% to 2.1 ± 3.6% (*p* < 0.001) with the slip reduction ratio 57.9 ± 43.9%, and the SL was increased from 8.3 ± 3.0° to 10.7 ± 3.6° (*p* = 0.008). Radiographic parameters at different stages of OLIF procedures were shown in Table 2 and Fig. 2.

**Table 2:** Radiographic parameters of segmental alignment at different stages of OLIF procedures

	Prior to cage insertion	Following cage insertion	Following posterior fixation	*P* Value	Comparison prior to Cage insertion and following cage insertion (P Value)	Comparison following cage insertion and following posterior fixation (*P* value)
Disc height (mm)	8.2 ± 1.6	11.8 ± 1.7	11.8 ± 2.3	< 0.001^*^	< 0.001^*^	1.000
Disc height-Anterior (mm)	9.6 ± 2.3	13.4 ± 2.1	13.7 ± 2.3	< 0.001^*^	< 0.001^*^	0.861
Disc height-Posterior (mm)	6.1 ± 1.9	9.1 ± 2.1	8.4 ± 1.8	< 0.001^*^	< 0.001^*^	0.254
Slip ratio (%)	11.1 ± 4.6	8.3 ± 4.4	2.1 ± 3.6	< 0.001^*^	0.020^*^	< 0.001^*^
Segmental lordosis (°)	8.7 ± 3.7	8.3 ± 3.0	10.7 ± 3.6	0.006^*^	1.000	0.008*

*Means statistically significant

**Fig. 3:**
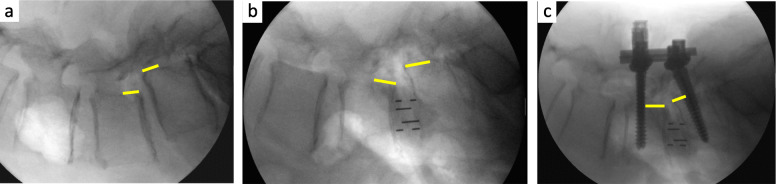
The case example of OLIF with open pedicle screw fixation for degenerative spondylolisthesis. a. Prior to cage insertion. Disc height (DH) 6.7 mm; Slip ratio 20.1%; Segmental lordosis (SL) 14.7°. b. Following cage insertion. DH 8.4 mm; Slip ratio 24.7%; SL 18.5°. c. Following posterior fixation. DH 8.4 mm; Slip ratio 6.5%; SL 23.8°

**Fig. 4:**
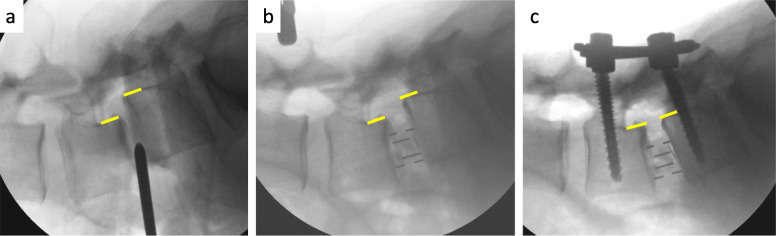
The case example of OLIF with percutaneous pedicle screw fixation for degenerative spondylolisthesis. a. Prior to cage insertion. Disc height (DH) 4.4 mm; Slip ratio 23.0%; Segmental lordosis (SL) 0.7°. b. Following cage insertion. DH 11.2 mm; Slip ratio 16.4%; SL 9.0°. c. Following posterior fixation. DH 12.7 mm; Slip ratio 0%; SL 17.4°

Two case examples of OLIF with open and percutaneous pedicle screw fixation were shown in Figs. 3 and 4.

The differences between the two groups of open and percutaneous techniques of pedicle screw fixation were analyzed. Among these five radiographic parameters, only DH-Anterior showed significant difference between two groups (*P* = 0.005) following ANOVA with multiple measures. The results of DH-Anterior prior to cage insertion and following posterior fixation were different between two groups (*P* = 0.019 and *P* = 0.012) following Student *t* test. The detailed results were shown on Table 3.

**Table 3:** Radiographic parameters of segmental alignment between open and percutaneous techniques of pedicle screw fixation

Radiographic parameters	Prior to cage insertion	Following cage insertion	Following posterior fixation
Disc height (mm)			
Open	8.7 ± 1.7^a^	11.9 ± 1.9	11.4 ± 2.4
Percutaneous	7.3 ± 1.2^a^	11.5 ± 1.3	12.5 ± 2.1
Total	8.2 ± 1.6	11.8 ± 1.7	11.8 ± 2.3
Disc height-Anterior^b^ (mm)			
Open	10.3 ± 2.3^a^	13.9 ± 2.2	14.3 ± 2.2^a^
Percutaneous	8.4 ± 2.0^a^	12.6 ± 1.5	12.3 ± 1.8^a^
Total	9.6 ± 2.3	13.4 ± 2.1	13.7 ± 2.3
Disc height-Posterior (mm)			
Open	6.4 ± 2.1	9.2 ± 2.1	8.5 ± 2.0
Percutaneous	5.5 ± 1.1	8.9 ± 2.1	8.2 ± 1.4
Total	6.1 ± 1.9	9.1 ± 2.1	8.4 ± 1.8
Slip ratio (%)			
Open	10.8 ± 4.4	8.3 ± 4.4	2.1 ± 3.7
Percutaneous	11.6 ± 5.1	8.3 ± 4.7	2.2 ± 3.4
Total	11.1 ± 4.6	8.3 ± 4.4	2.1 ± 3.6
Segmental lordosis (°)			
Open	9.5 ± 3.5	8.7 ± 3.2	10.9 ± 3.8
Percutaneous	7.1 ± 3.7	7.4 ± 2.5	10.4 ± 3.3
Total	8.7 ± 3.7	8.3 ± 3.0	10.7 ± 3.6

^a^Means comparison of open and percutaneous techniques showed statistically significant

^b^Means statistically significant following multivariate analysis between open and percutaneous techniques

Preoperative Visual Analogue Scale (VAS) for back pain was 4.8 ± 3.45, VAS for leg pain 6.1 ± 1.6, Japanese Orthopaedic Association (JOA) score 15.9 ± 6.1 and Oswestry Disability Index (ODI) 47.9 ± 20.1%. Postoperative VAS for back pain was 1.6 ± 1.5, VAS for leg pain 1.3 ± 1.5, JOA score 22.6 ± 5.3 and ODI 23.8 ± 17.6% at 3-month follow-up. Postoperative VAS for back pain was 0.8 ± 1.0, VAS for leg pain 1.1 ± 1.7, JOA score 24.1 ± 5.6, and ODI 14.2 ± 13.5 at 1-year follow-up. All the differences between preoperative and postoperative VAS, JOA score and ODI at 1-year follow-up were shown statistically significant by paired Student *t* tests. The treatment effects at 1-year follow-up were 3.7 (95% confidential interval [CI], 2.6–4.8) for VAS of back pain, 4.7 (95% CI, 3.8–5.5) for VAS of leg pain, 8.5 (95% CI, 5.8–11.2) for JOA score and 30.1 (95% CI, 24.7–37.3) for ODI.

The postoperative complications were also evaluated, 4 patients complained transient weakness of hip flexion and numbness over the anterior thigh which disappeared within 3 months. Six patients complained of residual neurological deficit which was not relieved at postoperative 1 year. No obvious endplate subsidence (> 2 mm subsidence) or cage migration or pedicle screw loosening were observed on the lumbar radiographs at postoperative 1 year.

## Discussion

In patients with degenerative spondylolisthesis, anterior displacement of inferior articular processes and osteophyte formation at superior articular process lead to lateral recess stenosis, while the displacement of the disc and thickening of ligamentum flavum cause central canal stenosis [3]. Choosing lumbar fusion for patients with symptomatic degenerative spondylolisthesis was challenged [12, 13], especially for degenerative spondylolisthesis without segmental instability. If lumbar fusion was necessary with the evidence of segmental instability, using mini-open oblique lateral approach, OLIF allows for large cage insertion which can result in reduction of disc bulging, the elongation of ligamentum flavum and thus enlarging lumbar spinal canal [6, 14, 15]. That’s why indirect neural decompression could be achieved through this method.

### The slip reduced by OLIF

The degree of slip negatively correlates with canal size and patients’ quality of life preoperatively [16, 17]. Although attempt for complete reduction of slip was not necessary in direct decompression procedure in terms of improving patient-reported outcomes [18], complete reduction of slip and restoring the normal anatomy of this segment can increase the canal size, meanwhile it can achieve indirect decompression in OLIF procedure with percutaneous pedicle screw fixation.

Sato et al [15] compared axial canal diameter, sagittal canal diameter and spinal canal cross-sectional area before and after OLIF procedures with posterior fixation for degenerative spondylolisthesis. Slip ratio was reduced from 14% preoperatively to 5% postoperatively and all those parameters of canal size were increased with slip reduction. As a result, reducing slip as much as possible can decompress the nerve impingement to the greatest extent, particularly necessary for indirect decompression in OLIF procedures.

Inserting large-size cage raises the disc height, hence stretches the ligamentous structures around the slipped level and reduces the slip. In this study, following cage insertion, disc height was improved from 8.2 mm to 11.8 mm, while the slip ratio was improved from 11.1 to 8.3% and slip reduction ratio was 25.6% on average, which meant one quarter of slip were reduced by stand-alone cage insertion. These findings supported the mechanism of slip reduction by cage insertion alone. However, residual slip of 8.3% limits the stand-alone technique in terms of capacity of indirect nerve decompression.

In this study, following posterior fixation, the slip was further reduced to 2.1%, slip reduction ratio was 57.9% on average, which meant greater than half of slip reduction was achieved by posterior fixation. Therefore, additional posterior fixation and reduction maneuver could reduce the slip to larger extent than stand-alone technique could.

### Segmental lordosis improved by OLIF

Segmental lordosis can be improved by insertion of cage with lordotic angle design [19]. The magnitude of improvement correlates with preoperative segmental lordosis and anteroposterior position of cage [20]. This current study, however didn’t show increase the segmental lordosis by cage insertion alone (8.7°to 8.3°). The likely cause was that the large preoperative segmental lordosis (8.7°) limited the capacity of anterior realignment due to tightness of anterior longitudinal ligament. Additional posterior fixation shortened the posterior column and further increased the segmental lordosis (10.7°) in this study, which indicated that posterior fixation could further improve segmental lordosis even if anterior realignment reached its limit.

### Stand-alone versus additional posterior fixation

Stand-alone cage insertion of OLIF procedure without posterior fixation is advocated by some surgeons [7, 8]. Several clinical results favoring standalone technique were reported in the literature [7, 21], and those favorable outcomes depend on inserting a large cage which can both achieve indirect decompression effect and provide instant stability by axial loading [6]. However, some drawbacks of stand-alone technique were shown during follow-ups. Cage subsidence and subsequent loss of correction may occur without posterior fixation [8]. The effect of indirect decompression was also decreased during follow-up in some patients undergoing stand-alone techniques [10]. A recent meta-analysis showed the reoperation rate and occurrence of cage migration was higher for standalone technique [9].

OLIF with posterior fixation can enhance the segmental stability, decrease the rate of cage subsidence and migration, and maintain the instant indirect decompression effect by cage insertion [9]. Additionally, as this study revealed, posterior fixation for patients with degenerative spondylolisthesis can further reduce the slip that maximizes the effect of indirect decompression, together with improvement of segmental lordosis. Therefore, OLIF with additional posterior fixation was recommended for patients with degenerative spondylolisthesis.

### Limitations

Although this study allows to demonstrate the changes of segmental alignment within the same procedure setting, this retrospective observational study has some limitations. Firstly, all the slip were Grade I spondylolisthesis (slip ratio: 3.5 to 23%) with most slipped levels located at the L4/5 level, even if the inclusion criteria included Grade I and II slips, which may constrain drawing conclusion for Grade II slip or other segments. Secondly, additional fixation did improve the segmental alignment together with favorable symptoms and disability improvements in short-term, however, whether the improved segmental alignment or the superiority of posterior fixation can be maintained is still uncertain in long-term. Thirdly, the result of sagittal alignment changes was derived from combined analysis of open and percutaneous pedicle screw fixation due to relatively small sample size. Open and percutaneous fixation probably result in difference alignment changes. However, separate analysis showed consistent outcome between two groups, making the combined analysis reasonable.

## Conclusions

Stand-alone cage insertion did have some degree of slip reduction and restoration of disc height. However, compared with stand-alone cage insertion, additional posterior fixation provides better segmental alignment improvement in terms of slip reduction and segmental lordosis in OLIF procedures in the treatment of lumbar degenerative spondylolisthesis.

## Data Availability

The data used to support the findings of this study are available from the corresponding author upon request.

## Abbreviations

OLIF: Oblique lumbar interbody fusion
DH: Disc height
SL: Segmental lordosis
